# Unraveling the Role of Drug-Lipid Interactions in NSAIDs-Induced Cardiotoxicity

**DOI:** 10.3390/membranes11010024

**Published:** 2020-12-29

**Authors:** Catarina Pereira-Leite, Marina Figueiredo, Kinga Burdach, Cláudia Nunes, Salette Reis

**Affiliations:** LAQV, REQUIMTE, Departamento de Ciências Químicas, Faculdade de Farmácia, Universidade do Porto, 4050-313 Porto, Portugal; marinacoimbra97@gmail.com (M.F.); kburdach@chem.uw.edu.pl (K.B.); cdnunes@ff.up.pt (C.N.); shreis@ff.up.pt (S.R.)

**Keywords:** diclofenac, naproxen, mitochondrial membranes, partition coefficient, membrane permeability, membrane structure

## Abstract

Cardiovascular (CV) toxicity is nowadays recognized as a class effect of non-aspirin nonsteroidal anti-inflammatory drugs (NSAIDs). However, their mechanisms of cardiotoxicity are not yet well understood, since different compounds with similar action mechanisms exhibit distinct cardiotoxicity. For instance, diclofenac (DIC) is among the most cardiotoxic compounds, while naproxen (NAP) is associated with low CV risk. In this sense, this study aimed to unravel the role of drug-lipid interactions in NSAIDs-induced cardiotoxicity. For that, DIC and NAP interactions with lipid bilayers as model systems of cell and mitochondrial membranes were characterized by derivative spectrophotometry, fluorometric leakage assays, and synchrotron X-ray scattering. Both DIC and NAP were found to have the ability to permeabilize the membrane models, as well as to alter the bilayers’ structure. The NSAIDs-induced modifications were dependent on the lipid composition of the membrane model, the three-dimensional structure of the drug, as well as the drug:lipid molar ratio tested. Altogether, this work supports the hypothesis that NSAIDs-lipid interactions, in particular at the mitochondrial level, may be another key step among the mechanisms underlying NSAIDs-induced cardiotoxicity.

## 1. Introduction

Therapy with nonsteroidal anti-inflammatory drugs (NSAIDs) has been associated with a high incidence of adverse effects, particularly if used in long-term treatments [1]. In 1938, Douthwaite and Lintott demonstrated that aspirin induces gastric damage [2], and later, the gastrointestinal (GI) toxicity was identified as a class effect of NSAIDs. The toxicity mechanisms of NSAIDs comprise not only their systemic effect of reducing the biosynthesis of gastroprotective prostaglandins, but also the topical actions of these drugs in the GI tract [3]. Among the latter actions, the NSAIDs-lipid interactions were found to be a key factor favoring the occurrence of adverse effects, with numerous contributions from our research group and others [4,5,6,7,8].

In the beginning of the 21st century, cardiovascular (CV) toxicity was associated with anti-inflammatory drugs for the first time, culminating in the withdrawal of rofecoxib and valdecoxib from the market in 2004 and 2005, respectively [9]. The evidence that cyclooxygenase-2 selective inhibitors were related to the occurrence of thrombotic events [10] raised questions about the CV safety of the entire class of NSAIDs. Nowadays, all non-aspirin NSAIDs are considered potential cardiotoxic agents by the regulatory agencies [11]. Nevertheless, different CV risks have been reported for distinct NSAIDs, with diclofenac belonging to the higher-risk group and naproxen belonging to the lower-risk group [12]. Mechanistic details are still awaited to clarify the differential cardiotoxic actions of NSAIDs.

Increasing evidence suggests that the manifestation of CV diseases, such as hypertension and sudden cardiac death, is related to alterations in lipid levels and membrane structure [13]. For instance, the fatty acid composition and the membrane fluidity of the erythrocytes of hypertensive patients was found to be disturbed in comparison with normotensive subjects [14,15]. Simultaneously, various NSAIDs were found to induce morphologic changes in red blood cells, including mefenamic acid, diclofenac, ibuprofen, naproxen, and aspirin [16,17,18,19,20]. In this sense, it is conceivable that NSAIDs-induced changes in biological membranes are an additional mechanism underlying the cardiotoxicity of these drugs. However, molecular details on drug-lipid interactions are awaited to eventually correlate the differential CV risk reported for each NSAID with the drug-induced alterations in lipid membranes.

**Figure 1:**
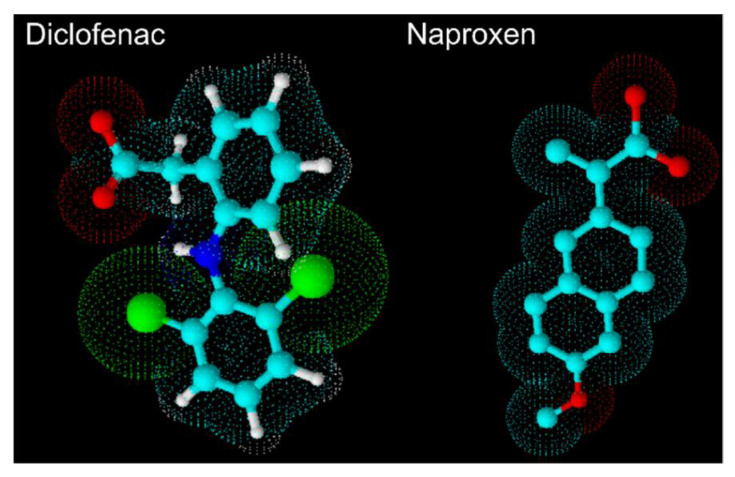
Three-dimensional structural formula of diclofenac and naproxen from ACD/ChemSketch 12.01.

In this work, we aimed to describe the effects of diclofenac (DIC) and naproxen (NAP) on the permeability and structure of lipid bilayers, used as simple model systems of cell and mitochondrial membranes. The rational beyond this study was based on the following points: (a) DIC and NAP (Figure 1) were chosen because they belong to the higher and lower risk groups (respectively) of NSAIDs to induce cardiotoxicity [12], (b) beyond cell membranes, mitochondrial membranes are of particular interest due to the high density of mitochondria existing in cardiomyocytes, which is crucial to maintain cardiac function; (c) cell death in the myocardium may occur due to alterations in the structure of both inner and outer mitochondria membranes [21]. Therefore, NSAIDs-lipid interactions were evaluated using 1-palmitoyl-2-oleoyl-*sn*-glycero-3-phosphocholine (POPC):cholesterol (80:20) as a model system of cell membranes, POPC:cardiolipin (85:15) as model system of the inner mitochondrial membrane, and POPC:phosphatidylinositol (85:15) as model system of the outer mitochondrial membrane. The lipid composition of membrane models was based on the specific lipid components of cell and mitochondrial membranes [22]. Molecular details on NSAID-lipid interactions were assessed by derivative spectrophotometry to evaluate the affinity of drugs for membrane models, fluorometric leakage assays to study the membrane permeabilizing activity of drugs, and synchrotron X-ray scattering to characterize the drugs’ effects on membrane structure. Overall, this study is another step towards unravelling the role of drug-lipid interactions in NSAIDs-induced cardiotoxicity.

## 2. Materials and Methods

### 2.1. Materials

Diclofenac sodium salt (DIC), naproxen sodium salt (NAP), 1-palmitoyl-2-oleoyl-*sn*-glycero-3-phosphocholine (POPC), Trizma^®^ base, sodium chloride, sodium hydroxide, potassium phosphate monobasic, ascorbic acid, 1,3,6,8-pyrenetetrasulfonic acid tetrasodium salt hydrate (PTS), methyl viologen dichloride hydrate (MV), Sephadex G-25^®^ medium, and nonaethylene glycol monododecyl ether (polidocanol) were supplied by Sigma-Aldrich Co. (St. Louis, MO, USA). Cholesterol from ovine wool (CHOL), 1′,3′-bis[1,2-dimyristoyl-sn-glycero-3-phospho]-glycerol (sodium salt) (TMCL), cardiolipin sodium salt from bovine hearth (CL), and L-α-phosphatidylinositol sodium salt from bovine liver (PI) was supplied by Avanti Polar Lipids, Inc. (Alabaster, AL, USA). Chloroform and methanol were obtained from VWR International S.A.S. (Fontenais-sous-Bois, France). Ammonium heptamolybdate tetrahydrate and hydrochloric acid were acquired from E. Merck (Darmstadt, Germany) and Fisher Scientific UK (Loughborough, UK), respectively. Perchloric acid was purchased by Riedel-de Haen (Seelze, Germany).

All assays were performed mimicking physiological conditions using Tris-HCl buffer (pH 7.4). It was prepared by dissolving Trizma^®^ base (10 mM) and NaCl (150 mM) in double deionized water (κ < 0.1 μS.cm^−1^) and adjusting the pH value using an HCl solution (1 M).

### 2.2. Preparation of Liposomes

Large unilamellar vesicles (LUVs) made of POPC, POPC:CHOL (80:20), POPC:TMCL(85:15), and POPC:PI (85:15) were prepared by the thin-film hydration method, in line with previously reported procedures [6,7]. Briefly, lipids were dissolved in chloroform:methanol (3:2, *v*/*v*), and lipid films were prepared by evaporating the organic solvents to dryness in a rotary evaporator coupled with an N_2_ stream at 40 °C. LUVs were finally produced by hydrating the lipid films with Tris-HCl buffer, followed by vigorous vortexing and extruding through 600 nm (5 times) and 100 nm (10 times) pore-sized polycarbonate filters (Nucleopore Track-Etch Membrane, Whatman) at 25 °C.

### 2.3. Derivative Spectrophotometry

The partition coefficient of DIC and NAP were determined by derivative spectrophotometry in LUVs:water systems, as reported elsewhere [6,7] with some modifications. Samples with increasing concentrations of LUVs (0–2 mM) and a fixed drug concentration (40 μM for DIC and 5 μM for NAP) were prepared. After vortexing, the samples were incubated at 37 °C for 30 min, before being transferred to 96-well plates. The absorption spectra (200–400 nm) were then acquired at 37 °C in a Synergy HT plate reader (BioTek Instruments, Inc., Bad Friedrichshall, Germany). Data analysis to estimate the partition coefficient values (*K_p_*) was performed using *K_p_* calculator [23], which ultimately considers Equation (1) to calculate *K_p_* using a nonlinear least-squares regression method, as detailed elsewhere [6]:

(1) $$D_{T}=D_{W}+\frac{bK_{p}\left[L\right]}{1+K_{p}\left[L\right]}$$

From the retrieved *K_p_* values, dimensionless log *D* values were calculated using log *D* = log(*K_p_*/*V_φ_*), considering the following lipid molar volumes (*V_φ_*): 0.756 L/mol for POPC [24], 0.681 L/mol for POPC:CHOL (80:20) [24], 0.987 L/mol for POPC:TMCL (85:15) [24,25], and 0.687 L/mol for POPC:PI (85:15) [26].

### 2.4. Fluorometric Leakage Assay

The effects of DIC and NAP on the bilayer permeability were assessed by fluorometric leakage assays using PTS and MV as fluorophore-quencher pair (respectively), using an adapted method of Manzini et al. [27]. Lipid films made of POPC:CHOL (80:20), POPC:TMCL (85:15), and POPC:PI (85:15) were hydrated with a PTS solution (1 mM) prepared in Tris-HCl buffer, and LUVs were prepared as described in Section 2.1. The non-loaded fraction of PTS was then separated from PTS-loaded LUVs by size exclusion chromatography, using Sephadex G-25^®^ medium as stationary phase and Tris-HCl buffer as mobile phase. Two sets of samples containing PTS-loaded LUVs, increasing concentrations of DIC or NAP (0–400 μM), and MV (1 mM) were prepared and incubated at 37 °C for 30 min. After that, a 15 μL aliquot of polidocanol solution (10% *v*/*v*) was added to one set of samples to completely disrupt the previously formed LUVs, while the other set of samples was treated with the same amount of Tris-HCl buffer. After transferring the samples to a 96-wells plate, their fluorescence intensity was measured at 37 °C in a Cytation 3 imaging reader (Biotek Intruments, Inc.), setting the excitation and emission wavelengths to 355 nm and 385 nm, respectively. The percentage of PTS leakage was determined as follows:

(2) $$Leakage\;\%=\frac{I_{0}-I_{t}}{I_{0}-I_{tot}}\times 100$$

where *I*_0_ stands for the fluorescence intensity of PTS in the absence of drug, *I_t_* is the fluorescence intensity of PTS in the presence of drug, and *I_tot_* is the fluorescence intensity of PTS after adding polidocanol. When necessary, the acquired fluorescence intensity values were corrected to discount the intrinsic fluorescence of drugs at the emission wavelength.

### 2.5. Inorganic Phosphate Analysis

The phospholipid concentrations of the collected aliquots of PTS-loaded LUVs after the size exclusion chromatography were determined by inorganic phosphate analysis, as previously reported [6,27], through the molybdenum blue reaction using ammonium heptamolybdate tetrahydrate as the source of molybdate, perchloric acid as a strong acid, and ascorbic acid as reductant. The blue-colored product was quantified by UV-Vis spectrophotometry (Jasco V-660, Pfungstadt, Germany) at 797 nm using a calibration curve method obtained with increasing quantities of potassium phosphate monobasic (0–100 nmol).

### 2.6. Synchrotron X-ray Scattering

Synchrotron X-ray scattering was used to evaluate the NSAIDs-induced alterations in the structure of phospholipid bilayers, as reported elsewhere [5,7]. Stacked bilayers made of POPC:CHOL (80:20), POPC:CL (85:15), and POPC:PI (85:15) in the absence and presence of increasing molar ratios of DIC and NAP (0–0.5) were prepared by co-dissolving lipid and drug in chloroform:methanol (3:2), evaporating the solvents at 40 °C under N_2_ stream, and leaving overnight under reduced pressure. These films were then hydrated with Tris-HCl buffer at 25 °C, vigorously vortexed, and transferred to X-ray transparent glass capillaries (1 mm diameter, Hilgenberg GmbH, Germany), which were sealed and left at 4 °C for at least one week before data acquisition. Small-angle X-ray scattering (SAXS) measurements were performed at the noncrystalline diffraction beamline of ALBA synchrotron (Cerdanyola del Vallès, Spain) at 37 °C, after 10 min of equilibration time, by recording 5 frames of 5 s of exposure. SAXS detectors were previously calibrated with silver behenate. The SAXS patterns are herein displayed as scattering intensity (arbitrary units) as a function of *q*-value (*q*). Long-range repeated distances (*d*) were estimated (*d* = 2π/*q*) from the peak maximum positions, while correlation lengths (*ξ*) were calculated from the corresponding full width at half maximum (*fwhm*, *ξ =* 4π^2^/*fwhm*). Peak maximum positions and *fwhm* were determined by fitting the Lorentzian function to the experimental Bragg peaks using OriginPro 8.5 Software (Northampton, MA, USA).

## 3. Results

### 3.1. Partition Coefficient

**Figure 2:**
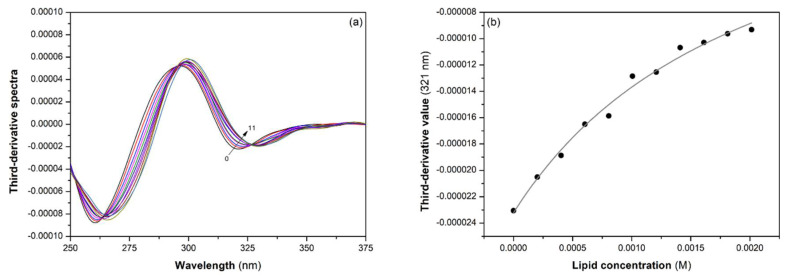
(**a**) Third-derivative spectra of DIC with increasing concentrations of POPC:CHOL (80:20) LUVS from 0 (0) to 2 mM (11) at 37 °C and pH 7.4; (**b**) Third-derivative values collected at 321 nm as a function of total lipid concentration. The gray line is the best fit for Equation (1).

The partition coefficient of DIC and NAP were determined at 37 °C and pH 7.4 by derivative spectrophotometry using POPC, POPC:CHOL (80:20), POPC:TMCL (85:15), and POPC:PI (85:15) LUVs. This assay consists of (a) recording the absorbance spectra of the drug with increasing concentrations of LUVs, (b) calculating the third-derivative spectra, and (c) plotting the third-derivative values (at 213 nm for NAP and 321 nm for DIC) versus LUVs concentration to calculate the partition coefficient of drugs using a nonlinear least-squares regression method to fit Equation (1) to the data. Data analysis performed for DIC to evaluate the partition coefficient in POPC:CHOL (80:20) LUVs is illustrated in Figure 2, as an example.

The log *D* values obtained from at least three independent assays are displayed in Table 1. The data indicated that the affinity of NAP and DIC to lipid bilayers depends on lipid composition. NAP displayed more affinity for POPC:CHOL and POPC:PI LUVs, as log *D* values obtained in these systems were superior to those obtained in POPC and POPC:TMCL LUVs. On the other hand, the affinity of DIC for POPC, POPC:CHOL, and POPC:PI LUVs were similar, while it was inferior for POPC:TMCL LUVS. Remarkably, the affinity of DIC and NAP for POPC LUVs were similar. However, the affinity of DIC was lower than that of NAP for all other lipid systems.

### 3.2. Membrane Permeabilizing Activity

**Figure 3:**
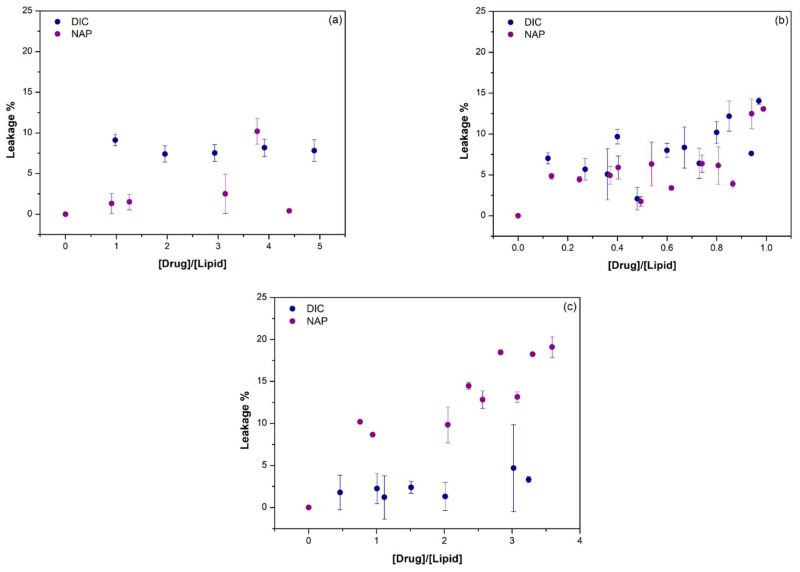
The effect of drug:lipid molar ratio on the PTS leakage from (**a**) POPC:CHOL (80:20), (**b**) POPC:TMCL (85:15), or (**c**) POPC:PI (85:15) LUVs at 37 °C and pH 7.4. DIC (blue circles) and NAP (purple circles) induced effects are presented as mean ± standard deviation of at least two replicates.

The permeabilizing activity of DIC and NAP in POPC:CHOL (80:20), POPC:TMCL (85:15), and POPC:PI (85:15) LUVs at pH 7.4 and 37 °C was assessed by fluorometric leakage assays, using PTS as fluorophore in the inner compartment of LUVs and MV as fluorescence quencher in the external aqueous medium. The maximum fluorescence intensity was obtained in the absence of drug, corresponding to a null leakage percentage. On the other hand, the minimum fluorescence intensity was obtained when polidocanol was added to the samples, as this detergent completely disrupts the vesicles, causing the maximum fluorescence quenching and the maximum leakage percentage. Considering the fluorometric data in the absence and presence of drugs, as well as upon polidocanol, the PTS leakage percentage induced by increasing concentrations of DIC and NAP was calculated through Equation (2) and is presented in Figure 3 as a function of drug:lipid molar ratio.

The permeabilizing activity of DIC and NAP was dependent not only on the lipid composition of LUVs, but also on the drug:lipid molar ratio. The leakage activity of DIC was mostly higher than NAP in POPC:CHOL LUVs, causing almost 10% of PTS leakage for all studied drug:lipid molar ratio. In contrast, the NAP-induced PTS leakage in POPC:PI LUVs was superior to that induced by DIC, ranging from 10 to 20% as a function of drug:lipid molar ratio. The effects of DIC and NAP on the membrane permeability of POPC:TMCL LUVs were mostly similar, ranging from 5 to 15%. In general, the highest PTS leakage percentages were observed once again for the highest drug:lipid molar ratios tested. Overall, these data suggest that both DIC and NAP caused alterations in the structure of lipid bilayers, which were further studied by synchrotron X-ray scattering measurements.

### 3.3. Effects on Membrane Structure

**Figure 4:**
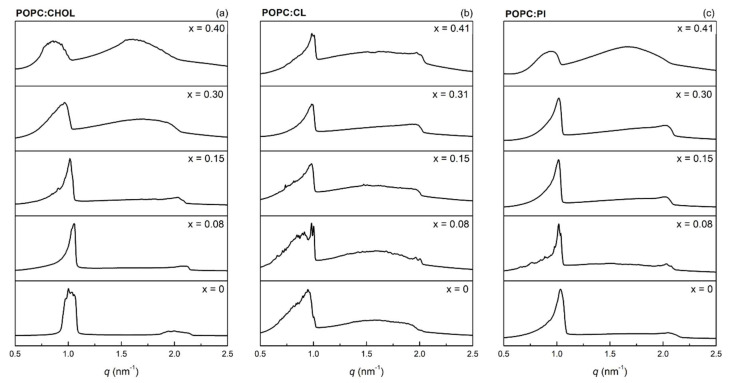
Average SAXS profiles of (**a**) POPC:CHOL (80:20), (**b**) POPC:CL (85:15), and (**c**) POPC:PI (85:15) at 37 °C and pH 7.4 as a function of DIC:lipid molar ratio (x).

**Figure 5:**
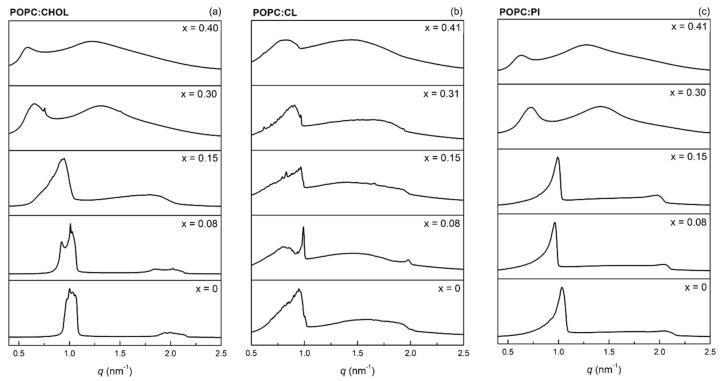
Average SAXS profiles of (**a**) POPC:CHOL (80:20), (**b**) POPC:CL (85:15), and (**c**) POPC:PI (85:15) bilayers at 37 °C and pH 7.4 as a function of NAP:lipid molar ratio (x).

The effects of DIC and NAP on the structure of POPC:CHOL (80:20), POPC:CL (85:15), and POPC:PI (85:15) bilayers were assessed at pH 7.4 and 37 °C by synchrotron X-ray scattering measurements. Drug-induced changes in the SAXS profiles of the studied bilayers are shown in Figure 4 and Figure 5, corresponding to DIC and NAP, respectively. Both POPC:CHOL and POPC:PI formed stack bilayers with a lamellar structure, as the *q*-values of second-order Bragg peaks (ca. 2 nm^−1^) are double those of first-order Bragg peaks (ca. 1 nm^−1^). In contrast, the incorporation of CL in POPC bilayers partially hampered the formation of stacked bilayers with lamellar structure, since a broad peak from 0.5 to 2 nm^−1^ was visible as background. Similar background signals were also observed upon the incorporation of high amounts of DIC and NAP irrespective of the lipid bilayer under study, showing that the incorporation of charged molecules disturbed the formation of stacked bilayers from multilamellar vesicles.

**Figure 6:**
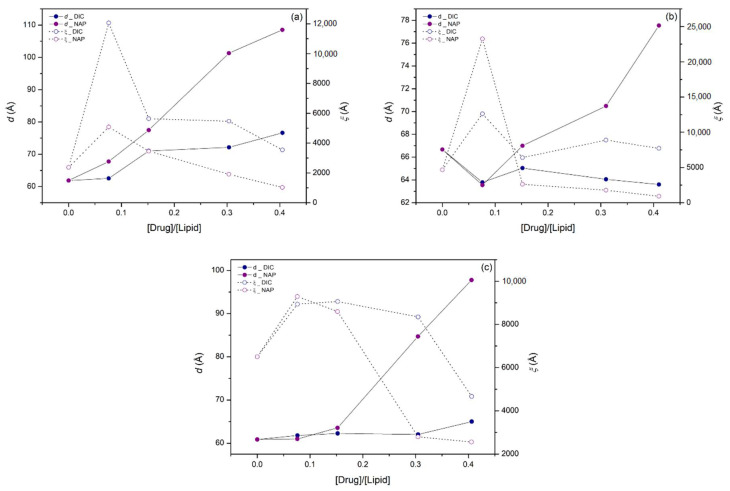
Long-range distance (*d,* solid circles, solid line) and correlation length (*ξ,* open circles, dashed line) corresponding to the most intense first-order Bragg peak of (**a**) POPC:CHOL (80:20), (**b**) POPC:CL (85:15), and (**c**) POPC:PI (85:15) bilayers at 37 °C and pH 7.4 as a function of drug:lipid molar ratio. DIC (blue circles) and NAP (purple circles) induced effects were calculated from the average SAXS profiles.

To better illustrate the effect of DIC and NAP on the bilayer thickness and its homogeneity, the most representative long-range distance (*d*) and the corresponding correlation length (*ξ*) were determined from the most intense first-order Bragg peak (Figure 6). Considering the POPC:CHOL bilayer, the higher the drug:lipid molar ratio, the higher the *d*-values, showing that both drugs increased the bilayer thickness and/or the thickness of water layers between lipid bilayers. It is noteworthy that the effect of NAP on *d-*values was more pronounced than that of DIC for all studied lipid bilayers. Indeed, only slight changes in *d*-values were found upon DIC incorporation into POPC:CL and POPC:PI bilayers, while the presence of NAP mostly caused the increase of *d*-values as a function of drug:lipid molar ratio. Regarding *ξ*-values, a prominent increase was observed for the lowest molar ratio of DIC or NAP in POPC:CHOL and POPC:CL bilayers. In contrast, a pronounced decrease of *ξ*-values was obtained for the highest molar ratio of DIC and NAP in POPC:PI bilayers, in line with the broader peaks observed in SAXS profiles, suggesting that the homogeneity of the bilayer thickness was reduced.

The effects of DIC and NAP at 0.08 molar ratio on POPC:CHOL and POPC:CL bilayers must be pointed out due to their singularity. Considering the former bilayer, the incorporation of DIC increased the lateral homogeneity of the POPC:CHOL bilayer, as a narrower Bragg peak (higher *ξ*-value) was observed. On the other hand, the incorporation of NAP into the POPC:CHOL bilayer caused the first-order Bragg peak to split in at least two peaks, showing that the bilayer may display narrower regions and thicker regions. A similar result was observed for DIC and NAP in POPC:CL bilayers, since the drug incorporation caused the division of the first-order Bragg peak into multiple narrower peaks with higher *ξ*-values, suggesting that both drugs induce an increment of lateral heterogeneity in this lipid system.

## 4. Discussion

The interactions of DIC and NAP with simple membrane models of cell membranes and mitochondrial membranes were evaluated herein. First, the affinity of drugs for the membrane models were assessed by derivative spectrophotometry, and the results showed that the drug’s partitioning depends not only on the drug tested, but also on the lipid composition of the membrane model. It is noteworthy that, to our knowledge, the partitioning of DIC and NAP was never reported for these membrane models. Despite that, the log *D* value found for DIC in POPC was similar to that previously reported in egg phosphatidylcholine [28,29]. This result was expected as this natural phospholipid is mainly constituted by palmitic acid and oleic acid, the fatty acids composing POPC. Interestingly, the affinity of DIC for the binary model systems reported in this study was inferior to that of NAP in all cases. This result may be related to the three-dimensional structure of both drugs (Figure 1), since the bulkier structure of DIC may decrease its ability to penetrate the lipid bilayers, while the planar structure of NAP may facilitate this process. This hypothesis is based on previously reported data concerning acidic NSAIDs location within phosphatidylcholine bilayers. Indeed, various experimental [5,6,7] and computational [30,31] data have been suggesting that acidic NSAIDs become anchored in the choline group of phosphatidylcholines due to electrostatic interactions, with the aromatic rings of the drug molecules inserted in the acyl chains region of the lipid bilayer. Thus, it is conceivable that the bulkier aromatic groups of DIC may function as a steric constraint to the drug partitioning. Regarding the lipid composition of the membrane models, DIC partitioning was only reduced in the POPC:TMCL system. TMCL is composed of two negatively charged moieties per lipid, as well as four saturated fatty acids, increasing the organization and the net charge of the membrane model. Thus, lower affinity could be expected for both DIC and NAP not only due to the higher order of the membrane model, but also because both drugs are negatively charged at pH 7.4. This latter conclusion was based on the *pK_a_* values of both drugs (4.0 and 4.2 for DIC and NAP, respectively) calculated using the MarvinSketch calculator (ChemAxon). However, the gathered data showed that the main constraints on the drug’s partitioning in POPC:TMCL bilayers were the membrane organization and the three-dimensional structure of drugs, since the log *D* value was only reduced in the case of the bulkier drug, namely DIC. Interestingly, the planar structure of NAP seems to be at the origin of its higher affinity to POPC:CHOL and POPC:PI bilayers. NAP may establish π-interactions with CHOL, increasing the drug affinity for the former model. On the other hand, PI is a natural phospholipid made of various unsaturated fatty acids, decreasing the order of the bilayer, probably facilitating the penetration of NAP in the existing voids due to its planar structure.

By interacting with the model membranes, DIC and NAP were also found to permeabilize the lipid bilayers in fluorometric leakage assays. The permeabilizing activity of these NSAIDs was dependent on the lipid composition of membrane models and the drug:lipid molar ratio. The permeabilizing activity of DIC was higher in POPC:CHOL bilayers, while NAP displayed the higher permeabilizing activity in POPC:PI bilayers. Moreover, the permeabilizing effect of both drugs was similar on POPC:CL bilayers. In a previous report, DIC was also found to have the ability to permeabilize other models of the inner mitochondrial membrane composed of DOPC:DOPE:CL (1:1:1) [28]. Moreover, nimesulide, another NSAID, also showed the ability to increase the permeability of POPC and DOPC:DOPE:TOCL (1:1:1) bilayers [32]. Despite the fact that no other membrane leakage studies have been reported in the literature, various studies have been showing the ability of DIC and NAP to fluidize lipid bilayers made of zwitterionic phospholipids [7,33] and negatively charged phospholipids [34]. By increasing the fluidity of membrane models, these drugs may increase their permeability, as reported in this study. The differential actions of DIC and NAP in the various membrane models may be related to their specific effects on the lateral organization of the lipid bilayers, which were further analyzed in this study by synchrotron X-ray scattering measurements.

SAXS data demonstrated that both DIC and NAP induce modifications in the bilayer thickness and in the lateral homogeneity of the membrane models under study. First of all, it is noteworthy that the accumulation of negative charges at the surface of the multilamellar vesicles hampered the formation of stacked bilayers, since the background signal typical of vesicles was observed upon the incorporation of CL and high amounts of DIC or NAP. Similar results were previously obtained with DIC, NAP, and other anionic NSAIDs in dimyristoylphosphatidylcholine bilayers [5,6,7,17,19], as the electrostatic repulsion due to the presence of anionic molecules on the surface of lipid vesicles may hinder the required reduction of curvature to form stacked bilayers. In line with the results reported herein for the POPC:CHOL system, both DIC and NAP were previously found to increase the bilayer thickness of phosphatidylcholine bilayers [7,17]. However, the incorporation of the lower amount of DIC under study increased the lateral homogeneity of the POPC:CHOL bilayer, while NAP in the same drug:lipid molar ratio increased the lateral heterogeneity of this bilayer, causing lateral phase separation. The latter result may be related to the putative higher affinity of NAP for CHOL than POPC due to the drug’s planar structure (as previously indicated to justify the higher affinity of NAP for POPC:CHOL bilayers), resulting in the accumulation of NAP near CHOL and decreasing the bilayer thickness in the areas where NAP and CHOL accumulate. The efficient interaction of NAP with CHOL in the POPC:CHOL bilayer may be responsible for the lower permeabilizing activity of NAP in comparison with DIC, since the latter drug may cause imperfections in the bilayer structure more easily due to its bulkier structure, leading to the enhancement of the bilayer permeability.

Concerning the drugs’ effects on mitochondrial membrane models, NAP was found by SAXS experiments to induce more pronounced effects than DIC both on the thickness and the lateral homogeneity of the POPC:CL and POPC:PI bilayers. It is remarkable that DIC only slightly altered the SAXS profile of POPC:PI bilayers, the same membrane model system on which this drug did not pronouncedly enhance membrane permeability. On the other hand, the most pronounced alterations induced by NAP on membrane structure were observed in POPC:PI bilayers, which was the membrane model on which this drug induced the most remarkable increase of membrane permeability. It is noteworthy that the increase of membrane permeability of mitochondria underlies the cell death in the myocardium, leading eventually to CV dysfunction [21]. In line with the results found in this study using simple membrane models, it was recently reported that both DIC and NAP are able to induce mitochondrial permeability transition in isolated mitochondria [35]. Altogether, these results indicate that both drugs are potential cardiotoxic agents and that the NSAIDs-lipid interactions may contribute to the CV toxicity associated with NSAIDs therapy.

Although this study has provided vital initial indications to eventually correlate the drug-lipid interactions with the cardiotoxicity of NSAIDs, the data obtained herein did not justify the differential CV risk associated with the clinical use of DIC and NAP. Indeed, a meta-analysis of observational studies indicated that the CV risk associated with the use of NAP is lower than that of DIC [12]. Therefore, further studies are awaited to distinguish the cardiotoxicity mechanisms of these drugs. It is noteworthy that biological membranes are complex and dynamic systems presenting lateral and cross-sectional asymmetry [13,36], which were not mimicked by the model systems used in this study. Thus, it is crucial to understand the impact of using more complex membrane models, for instance, by considering a higher diversity of lipids and asymmetric leaflets. These further studies combined with the data presented herein will be essential to ultimately ruling in or out the role of drug-lipid interactions as an additional cardiotoxicity mechanism of NSAIDs.

## 5. Conclusions

This initial investigation on the impact of DIC and NAP in simple model systems of cell membranes and mitochondrial membranes showed that both drugs can interact with lipid bilayers and change their permeability and structure. Since the alteration of membrane permeability and structure seems to be a general feature of CV dysfunction, this report gives initial support to the membrane hypothesis underlying the cardiotoxicity of NSAIDs. Nevertheless, further investigations are needed with more complex membrane models to comprehensively clarify the role of drug-lipid interactions in NSAIDs-induced cardiotoxicity and to eventually correlate the drug effects at the membrane level with the known CV risk of each NSAID.

## Figures and Tables

**Table 1:** Partition coefficient, expressed as log *D*, of NAP and DIC at 37 °C and pH 7.4 according to the lipid composition of LUVs.

LUVs	NAP ^1^	DIC ^1^
POPC	3.0 ± 0.1	2.9 ± 0.2
POPC:CHOL (80:20)	3.4 ± 0.1	2.8 ± 0.1
POPC:TMCL (85:15)	3.1 ± 0.1	2.5 ± 0.1
POPC:PI (85:15)	3.6 ± 0.1	2.9 ± 0.1

^1^ Values are expressed as mean ± standard deviation (*n* ≥ 3).

## Data Availability

The data presented in this study are available in this article.
